# Quantitative cistern effacement and reduced gray to white matter ratio for prognostication in early brain computed tomography of patients with cardiac arrest

**DOI:** 10.1016/j.heliyon.2023.e23741

**Published:** 2023-12-16

**Authors:** Jinsung Kim, Jae Hoon Lee

**Affiliations:** a Department of Emergency Medicine, Dong-A University College of Medicine, Busan, South Korea

**Keywords:** Out-of-hospital cardiac arrest, Multidetector computed tomography, Magnetic resonance imaging, Prognosis

## Abstract

**Background:**

The impact of cerebral edema on brain cells and ventricles in cardiac arrest patients can manifest as effacement of cortical sulci, diminished ventricle size, altered gray matter to white matter ratio (GWR), and increased optic nerve sheath diameter (ONSD) in brain CT scans. However, a complete investigation of GWR in whole lobes, quantitative cistern size, and comprehensive comparison of various brain CT parameters has not been conducted. This study aimed to comprehensively compare various early brain CT parameters along with conventional significant variables in relation to poor neurological outcome and diffuse cortical necrosis.

**Methods:**

This retrospective study included 86 adult patients with cardiac arrest who underwent brain CT/MRI. GWRs, the distance of the posterior ambient cistern, and ONSD in early brain CT and regions of interest (ROIs) in brain MRI were measured and analyzed along with clinical characteristics.

**Results:**

ROIs in the putamen and parietal white matter showed significant differences (p = 0.05, p = 0.022, respectively). The distance of the posterior ambient cistern and the GWR of the putamen and parietal white matter were newly developed predictors that were not used previously and demonstrated a significant correlation with the presence of diffuse cortical necrosis (OR 0.4, p = 0.006, AUC 0.637; OR 0.478, p = 0.02, AUC 0.603, respectively) or poor neurological outcomes (AUC 0.637, AUC 0.603, respectively), but were not more significant than pupil reflex (OR 0.06, p < 0.001). ONSD was not significantly associated with the outcomes.

**Conclusions:**

Quantitative cistern effacement and reduced GWR of the putamen and parietal white matter in early brain CT measurements of cardiac arrest patients were promising predictors in early brain CT for prognostication, but compared with clinical characteristics, the clinical significance of the CT predictors was not considerable. The relationship and clinical significance between the parameters in early brain CT and the outcomes might have to be separately considered.

## 1 Introduction

Hypoxic ischemic brain injury ensues following cardiac arrest, with primary and secondary injury mechanisms inducing brain damage [1]. Among the secondary brain injuries, cerebral edema, characterized by vasogenic and cytotoxic edema can lead to increased intracranial pressure (ICP), decreased cerebral perfusion pressure, decreased cerebral blood flow, herniation, and ultimately, brain death [2]. Cerebral edema primarily arises from cellular swelling over the first day after cardiac arrest [3]. The impact of cerebral edema on brain cells and ventricles can manifest as effacement of cortical sulci, diminished ventricle size, altered gray matter to white matter ratio (GWR), and increased optic nerve sheath diameter (ONSD) in brain CT scans [4,5]. Several predictors of poor neurological outcomes in cardiac arrest patients have been identified: sulcal effacement was associated with poor outcome, poor disposition, in-hospital mortality, and intensive care unit mortality [6]; reduced lateral ventricle area predicted poor neurological outcome with an AUC of 0.66 [7]; GWRs, as determined through a systemic review, were correlated with unfavorable neurological outcome [8]; and ONSD showed significant predictive value for poor neurological outcome with an AUC of 0.931 [5].

Brain computed tomography (CT) performed at an early stage has been conducted to discern the causes of cardiac arrest in patients who have experienced cardiac arrest. This brain CT has also been used for prognostication. Recently, the presence of a marked reduction in the gray matter to white matter ratio GWR on brain CT within 72 h after the restoration of spontaneous circulation (ROSC) with other prognostic modalities was suggested as representing a specific modality, although it was not high recommended [4]. The signs of diffuse and extensive hypoxic-ischemic brain injury, including an effacement of cortical sulci, reduced ventricle size, and reduced GWR, were suggested as one of 6 criteria for prognostication in this guideline.

The discriminative power of brain CT over 24 h after cardiac arrest became more powerful than that within 24 h after cardiac arrest [9,10]; however, routine brain CT within 24 h after cardiac arrest for prognostication was suspected of being using appropriately as a prognostic modality because previous studies had to resolve several issues. The following issues were present in these previous studies: (1) the GWRs of frontal, parietal, temporal, and occipital gray and white matter (other than basal ganglia) were not investigated overall, such as the apparent diffusion coefficient (ADC) values in brain magnetic resonance imaging (MRI); (2) the effacement of the sulcus or cistern has not been quantitatively measured; (3) the association between predictors of early brain CT (within 24 h) and poor neurologic outcome has shown different and controversial results [5,8,11,12]; and (4) the clinical significance was obscure because parameters in early brain CT for prognostication were not compared with each other or with other significant variables for prognostication.

Therefore, this study aimed to comprehensively compare various early brain CT parameters, including GWR, quantitative cistern size, and optic nerve sheath diameter (ONSD), along with conventional significant variables including pupil and corneal reflex in relation to poor neurological outcome and diffuse cortical necrosis.

## 2 Methods

### 2.1 Study design and setting

This retrospective observation cohort study was conducted by using data collected from patients with cardiac arrest in a tertiary teaching hospital from January 2019 to December 2022. The following inclusion criteria were used for this study: patients older than 18-years-old; patients with cardiac arrest who underwent targeted temperature management; patients with an unconscious mental state (Glasgow Coma Scale score <8) after the restoration of spontaneous circulation; and patients who underwent brain computed tomography (CT) on admission and brain magnetic resonance imaging (MRI) at 3 days after admission. The following exclusion criteria were used: numerous artifacts observed on brain CT or MRI (n = 4); brain CT being replaced by neck CT or not performed (n = 6); and brain CT on admission or brain MRI at 3 days after admission being delayed over 24 h (n = 7). A total 86 patients were included in the analysis. To minimize bias, we ensured that the results of patients' prognoses were not disclosed to the investigators. Additionally, to reduce measurement errors, the same investigator conducted measurements of brain CT and MRI parameters using a consistent method. This approach helped maintain uniformity and accuracy in our data collection process. This study was approved by the institutional review board of our hospital (DAUHIRB-16-079). In accordance with approved guidelines, patient data were anonymized and analyzed.

### 2.2 Clinical characteristics and outcome assessment

Data on age, sex, witnessed cardiac arrest, bystander cardiopulmonary resuscitation (CPR), presence of ventricular tachycardia or fibrillation, time from arrest to CPR start, CPR duration, and neurological examinations (such as the pupil reflex and corneal reflex) were collected on admission. The primary end point of this study included diffuse cortical necrosis on brain MRI at 3 days after admission and the secondary end point included cerebral performance category scores at 3 months after discharge. Cerebral performance category scores of 1 or 2 were considered to indicate a good prognosis, and the outcome was assessed via the investigation of medical records or a standardized telephone call by investigators.

### 2.3 Setting of brain MRI and measurement of MRI parameters

**Fig. 1:**
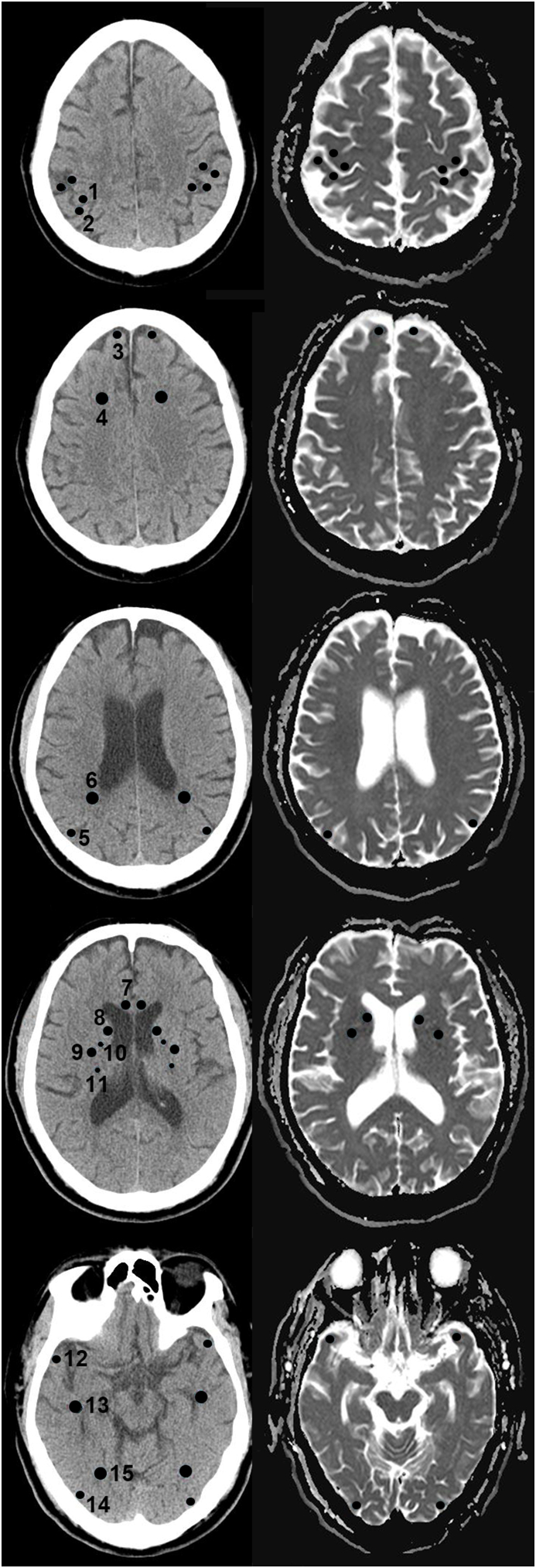
ROI regions in brain CT and MRI. (1) Precentral cortex, (2) postcentral cortex, (3) frontal cortex, (4) frontal white matter, (5) parietal cortex, (6) parietal white matter, (7) corpus callosum, (8) caudate nucleus, (9) putamen, (10) anterior limb of internal capsule, (11) posterior limb of internal capsule, (12) temporal cortex, (13) temporal white matter, (14) occipital cortex, and (15) occipital white matter.

A 1.5-T system (Signa Excite; General Electric, Milwaukee, WI, USA) with echo planar capability was used for the assessment of prognosis. The ADC maps were automatically generated. Only diffusion-weighted images (DWIs) data and ADC maps were analyzed in this study. The MRIs were reviewed on a standard picture archiving and communication system (PACS) workstation. Additionally, the ADC value of each pixel was constantly displayed on a region of interest (ROI). The ROIs were positioned on the areas with a minimum ADC on the predefined locations of the ADC maps to produce ADC values for each brain region (Fig. 1). Subsequently, the ADC measurements from both sides of the brain were averaged. ROI sizes were observed to vary by region when using 3–4 mm^2^ for all of the gray matter regions. The ROIs on ADC maps were measured at the precentral, postcentral, frontal cortex, parietal cortex, caudate nucleus, putamen, temporal cortex, and occipital cortex area; however, the mean ADC value was calculated by using only the value of the precentral, postcentral, frontal, parietal, temporal, and occipital cortex areas.

Diffuse cortical necrosis for anoxic brain injury was defined as definite high signal intensities on DWIs and low signal intensities on the predefined brain cortex of ADC maps. The presence of this parameter was compared with the mean ADC value by the ROI. Focal infarctions were not included in the definition of diffuse cortical necrosis.

### 2.4 Setting of brain CT and measurement of CT parameters

Noncontrast CT (SOMATOM Definition AS + scanner; Siemens, München, Deutschland) was used for the assessment of prognosis following standardized protocols with noncontrast 5 mm contiguous slices parallel to the orbital floor from the base of the skull to the vertex. We investigated brain CT parameters including GWR, effacement of sulcus, the ratio of ONSD to eyeball transverse diameter (ETD), and the reduced size of ventricles or cisterns in relation to poor neurological outcome in previous studies. Among these parameters, the GWRs in each brain area, the size of the posterior ambient cistern (which can be conveniently measured), and the ratio of ONSD to ETD were selected, but effacement of sulcus was excluded due to its subjective nature.

Circular ROIs were used to measure Hounsfield units (HUs) on each side and average values were recorded. Subsequently, the ROI measurements from both sides of the brain were averaged. GWR was defined as ROI ratio in gray and white matter of each brain area. ROI sizes were observed to vary by region by using 4–6 mm^2^ for the precentral, postcentral, frontal, parietal, temporal, and occipital cortex areas; in addition, they varied by using 30–40 mm^2^ for the frontal, parietal, temporal, and occipital white matter areas and by using 5–9 mm^2^ for the genu of the corpus callosum, caudate, and putamen; moreover, they varied by using 2–5 mm^2^ for the internal capsules. Furthermore, we measured the ROIs of the precentral and postcentral cortex at the level of high convexity; the frontal cortex and white matter at the level of the centrum semiovale; the parietal cortex and white matter at the level of the corona radiate; the genu of the corpus callosum, caudate nucleus, putamen, anterior limb, and posterior limb of the internal capsules at the level of the basal ganglia; and the temporal and occipital cortex and white matter at the level of the mid-brain (Fig. 1). In addition, we calculated and compared the mean ROIs of the precentral, postcentral, frontal, parietal, temporal, and occipital cortex areas; the mean GWRs by using the ROIs of the frontal, parietal, temporal, occipital cortex, and white matter areas; and the GWRs of the basal ganglia.

**Fig. 2:**
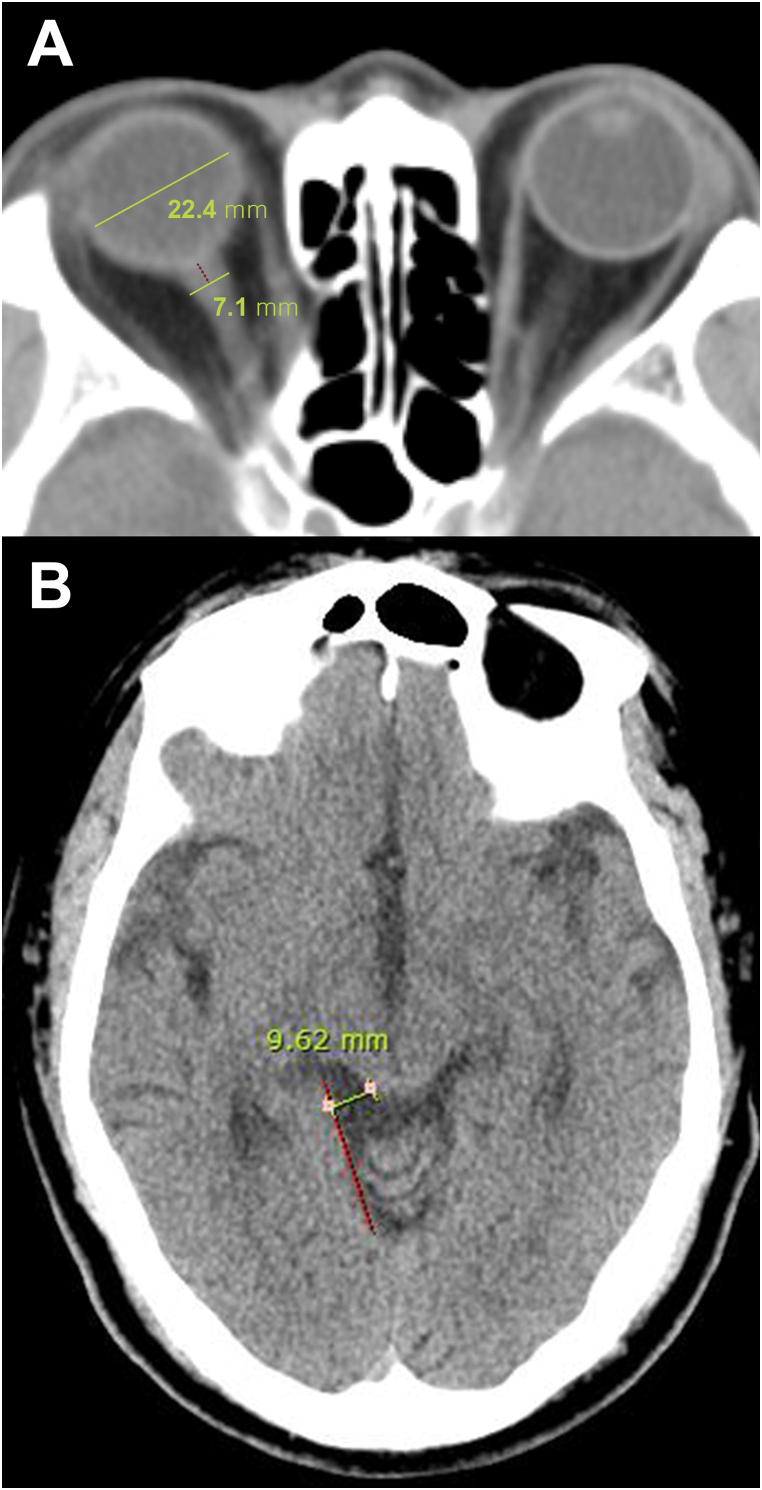
Measurement. of ONSD/ETD and the distance of the posterior ambient cistern. A: ONSD was measured at 3 mm behind the retina. B: The distance of the posterior ambient cistern was defined as the vertical distance from the extension line where the superior vermis and the occipital lobe meet to the superior colliculus of the midbrain.

The ONSD and eyeball transverse diameter (ETD) were measured on a PACS workstation using initial brain CT images. The window parameters included the chest/abdomen window (width: 300, level: 10). The selected image was magnified fourfold on the particular image slice showing the largest diameter of the optic nerve sheath (Fig. 2A). The ONSD was measured at a distance of 3 mm behind the globe below the sclera and the ETD was measured as the longest diameter of the eyeball.

There are several important subarachnoid cisterns, including the quadrigeminal, suprasellar, and prepontine cisterns; however, the quadrigeminal cistern was selected to measure the level of cistern effacement because its measurement was anatomically convenient. Posterior ambient cistern effacement was quantitatively measured as the vertical distance from the extension line where the superior vermis and the occipital lobe meet to the superior colliculus of the mid-brain at the level of the quadrigeminal cistern (Fig. 2B).

### 2.5 Statistical analysis

Continuous variables were shown as medians (IQR) and were analyzed by using the Mann–Whitney *U* test. The Fisher's exact test was used to compare the categorical variables. Significant variables were standardized and analyzed to estimate the risks of the outcomes via binary logistic regression, allowing for covariates. Moreover, a receiver operating characteristic analysis was used to compare the performance of the parameters. The performances of parameters for the primary and secondary outcomes were compared. A *P* value of <0.05 was considered to be statistically significant.

## 3 Results

### 3.1 Baseline characteristics

Among the 86 patients who experienced cardiac arrest, 46 patients (53.5 %) had a poor neurological outcome at the 3-month follow-up and diffuse cortical necrosis was observed in 36 patients (41.9 %). The distribution of cardiac arrest causes was as follows: 27 patients with acute coronary disease (31.4 %); 17 patients with myocardial disease (19.8 %); 4 patients with arrhythmia (4.7 %); 14 patients with airway obstruction (16.3 %); 5 patients with sepsis (5.8 %); 2 patients with drug intoxication (2.3 %); 2 patients with drowning (2.3 %); 3 patients with hypovolemia (3.5 %); 9 patients with unknown causes; and others with causes such as pulmonary embolism, hormonal disturbance, and cerebral infarction. In patients with cardiac arrest, various conventional variables were significantly correlated with diffuse cortical necrosis, including witness arrest, bystander CPR, shockable rhythm, time from arrest to CPR start, CPR duration, pupil reflex, corneal reflex, and history of previous cardiac/malignant/psychiatric diseases (Table 1).

**Table 1:** Baseline characteristics.

	All	Normal brain	DCN	*p*-value
Age, years	62 (52, 73)	62.5 (52, 73.5)	61.5 (50.5, 70.8)	0.697
Male, n (%)	57 (66.3)	35 (70)	22 (61.1)	0.489
Witness arrest, n (%)	71 (82.6)	46 (92)	25 (69.4)	0.009
Bystander CPR, n (%)	51 (59.3)	36 (72)	15 (41.7)	0.007
Shockable rhythm, n (%)	39 (45.3)	31 (62)	8 (22.2)	<0.001
Time from arrest to CPR start, minutes	5 (0.75, 10))	2.5 (0, 5)	8.5 (3, 14.3)	<0.001
CPR duration, minutes	20 (10, 31.5)	12 (7.75, 27.5)	25 (18.5, 40.3)	<0.001
Previous diseases, n (%)				
Hypertension	37 (43)	24 (48)	13 (36.1)	0.377
Diabetes mellitus	28 (32.6)	15 (30)	13 (36.1)	0.643
Cardiac disease	26 (30.2)	20 (40)	6 (16.7)	0.031
Arrhythmia	7 (8.1)	5 (10)	2 (5.6)	0.694
Brain disease	13 (15.1)	5 (10)	8 (22.2)	0.138
Kidney disease	11 (12.8)	6 (12)	5 (13.9)	1
Lung or liver disease	6 (7)	2 (4)	4 (11.1)	0.232
Malignancy	9 (10.5)	2 (4)	7 (19.4)	0.031
Psychiatric disease	13 (15.1)	3 (6)	10 (27.8)	0.012
Pupil reflex, n (%)	54 (62.8)	42 (84)	12 (33.3)	<0.001
Corneal reflex, n (%)	27 (31.4)	26 (52)	1 (2.8)	<0.001

DCN, diffuse cortical necrosis.

### 3.2 MRI and CT parameters

**Fig. 3:**
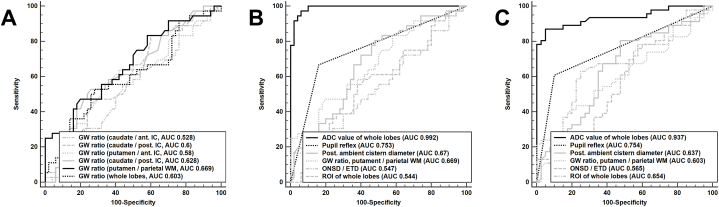
Prognostic performances of brain CT parameters. A: Performance of GWR on the basal ganglia. B: Performance of significant parameters for the presence of diffuse cortical necrosis. C: Performance of significant parameters for poor neurological outcomes.

The cutoff values of the mean ADC value from representative brain cortices for diffuse cortical necrosis and poor neurological outcomes were 677.8 mm^2^/s and 690 mm^2^/s, respectively. The cutoff value of the mean ROI value from representative brain cortices for diffuse cortical necrosis and poor neurological outcomes was 41.4 HU for both diffuse cortical necrosis and poor neurological outcomes; however, the discriminative powers only involved AUCs of 0.544 and 0.654, respectively. In Table 2, the mean ROI value was not significantly associated with the outcomes (p = 0.484), and the mean GWR from the predefined brain cortices and medullas also showed no correlation with the outcomes (p = 0.103). The previous criteria of GWRs on the basal ganglia exhibited low performance (Fig. 3A), and the ROI values on the basal ganglia were not significantly linked to the presence of diffuse cortical necrosis (other than the ROI of the putamen) (Table 2). Interestingly, the ROI value in the parietal white matter was significantly associated with the presence of diffuse cortical necrosis (Table 2).

**Table 2:** Prognostic factors from brain computed tomography for diffuse cortical necrosis.

	All	Normal brain	DCN	*p*-value
ROI in precentral cortex	40.5 (38.8, 44.1)	41 (38.7, 44.5)	40 (38.8, 43.5)	0.36
ROI in postcentral cortex	40.8 (38.8, 43.6)	41 (38.4, 43.5)	40.5 (38.8, 43.9)	0.951
ROI in frontal cortex	45 (41, 48)	33.5 (31.9, 35.5)	45 (41.5, 47.5)	0.951
ROI in frontal white matter	33.5 (32, 35.5)	33.5 (31.9, 35.5)	33.5 (32.5, 35.4)	0.765
GW ratio in the frontal lobe	1.38 (1.26, 1.46)	1.39 (1.26, 1.48)	1.36 (1.26, 1.43)	0.386
ROI in parietal cortex	41.5 (39.5, 44.1)	41.5 (39.5, 44.5)	41.5 (39.5, 43.9)	0.596
ROI in parietal white matter	31.5 (30, 33.5)	30.8 (29.5, 44.5)	32 (30.6, 34.5)	0.022
GW ratio in the parietal lobe	1.34 (1.23, 1.44)	1.37 (1.28, 1.46)	1.29 (1.19, 1.42)	0.037
ROI in Genu of CC	33 (30, 35)	33 (30, 35.25)	32.5 (31, 34)	0.741
ROI in caudate nucleus	38 (35, 40)	38 (34.8, 41)	37.5 (35.3, 39)	0.455
ROI in putamen	38 (34.8, 40)	38 (36, 40)	37 (34, 39)	0.05
ROI in anterior IC	35 (33, 37)	35 (33, 37.3)	35 (32, 37)	0.576
ROI in posterior IC	31.5 (30, 33.25)	31 (30, 33)	32 (30, 34)	0.396
ROI in temporal cortex	40.5 (37.9, 43.5)	41.3 (38.3, 43.6)	40 (37.6, 42)	0.266
ROI in temporal white matter	34 (32.5, 35.5)	34.3 (32, 35.5)	34 (32.5, 36.5)	0.38
GW ratio in the temporal lobe	1.18 (1.09, 1.29)	1.2 (1.12, 1.33)	1.15 (1.07, 1.24)	0.071
ROI in occipital cortex	41 (39, 45)	41 (39, 45)	41.3 (38.6, 45.3)	0.948
ROI in occipital white matter	34 (32, 36.1)	33.3 (31.5, 36)	34 (33, 36.5)	0.11
GW ratio in the occipital lobe	1.21 (1.14, 1.31)	1.22 (1.14, 1.36)	1.21 (1.12, 1.3)	0.413
Mean ROI of whole cortex	41.6 (39.8, 43.8)	41.8 (40.2, 43.8)	41.3 (39.8, 43.9)	0.484
GW ratio of whole lobes	1.28 (1.21, 1.34)	1.29 (1.22, 1.37)	1.24 (1.17, 1.34)	0.103
Right ONSD	6.04 (5.07, 6.7)	5.95 (5.03, 6.71)	6.11 (5.23, 6.76)	0.484
Left ONSD	6.16 (5.59, 6.73)	6.16 (5.81, 6.63)	6.23 (5.37, 6.96)	0.575
ONSD/ETD	0.25 (0.23, 0.28)	0.25 (0.23, 0.28)	0.26 (0.23, 0.29)	0.462
The distance of right PAC	3.82 (2.31, 5.38)	4.53 (2.51, 5.6)	3.36 (1.84, 4)	0.002
The distance of left PAC	3.58 (2.15, 5.26)	4.1 (2.34, 5.77)	2.68 (1.89, 4.52)	0.019
The distance of mean PAC	3.67 (2.15, 5.03)	4.71 (2.48, 5.72)	2.97 (1.9, 4.28)	0.007

DCN, diffuse cortical necrosis; ROI, region of interest; GW, gray matter/white matter; CC, corpus callosum; IC, internal capsule; ONSD, optic nerve sheath diameter, ETD, eyeball transverse diameter; PAC; posterior ambient cistern.

The ONSD and ONSD regarding the size of the eyeball were not associated with the presence of diffuse cortical necrosis or poor neurological outcomes. However, the reduced distance of the posterior ambient cistern denoting cistern effacement demonstrated a strong association with the outcomes (Table 2).

The pupil reflex was the most powerful predictor of baseline characteristics for prognostication and the odds ratios (OR) of the CT parameters that showed a significant correlation with the presence of diffuse cortical necrosis were not superior to those of the pupil reflex (Table 3). Among CT parameters, the performance of the distance of the posterior ambient cistern and the GW ratio (putamen/parietal white matter) for the presence of diffuse cortical necrosis and poor neurological outcomes showed AUCs of 0.6–0.67, whereas that of the pupil reflex was 0.753 or 0.754 (Fig. 3B and C).

**Table 3:** Multivariable regression analysis for diffuse cortical necrosis.

Standardized variables	Odds ratio	95 % CI	*p*-value
Pupil reflex	0.06	0.016–0.224	<0.001
The distance of posterior ambient cistern	0.4	0.2086–0.769	0.006
GW ratio (putamen/parietal white matter)	0.478	0.256–0.891	0.02
ONSD/ETD	5.614	0.721–43.709	0.099

## 4 Discussion

Among the examined CT parameters, the distance of the posterior ambient cistern was most associated with the presence of diffuse cortical necrosis, followed by the GWR of the putamen and parietal white matter. However, abnormal pupil reflex had a higher risk of poor outcomes than the CT parameters.

In brain CT within 24 h, it is well-known that cistern effacement is associated with poor outcomes in pediatric patients with cardiac arrest [13,14]. Ambient cistern effacement (34 of 50 poor outcome patients, AUC 0.804) had greater power in prognostication than GWR of the basal ganglia (AUC 0.744), which was similar to our data in a previous study [13]; however, basilar cistern effacement (13 of 29 poor outcome patients, AUC 0.68) showed less power than GWR (AUC 0.82) in another study [14]. The prognostic performance of posterior ambient cistern effacement in our data was smaller (AUC 0.637) than that in pediatric patients with cardiac arrest. The reason for this effect is likely because cardiac arrest patients at a younger age may have a better performance for prognostication than older patients [15]. In adult patients with cardiac arrest, cistern effacement in early brain CT has rarely been researched. A previous study demonstrated that basal cistern effacement was correlated with brain death after cardiac arrest (78 of 147 brain death patients) [16]. Moreover, basal cistern effacement was explained with a case with cardiac arrest in the form of case report in another study [17]. In particular, the quantitative measurement of cistern effacement has not been previously examined in pediatric or adult patients with cardiac arrest. Quantitative posterior ambient cistern effacement was the best predictor for poor neurological outcomes in early CT of cardiac arrest patients. The reappraisal of the clinical significance of quantitative cistern effacement based on our results is needed.

GWR on the basal ganglia has been mainly investigated; however, some studies have reported GWR in brain cortices, such as the frontal cortex and cerebellum [18,19]. Interestingly, the GWR in the parietal, temporal, and occipital lobes has rarely been evaluated. Only one study has evaluated the GWR with the inclusion of the parietal, temporal, and occipital lobes [20]. The corpus callosum and corona radiata between the measured white matter demonstrated lower HU values (25 HU, p = 0.01; 25.9 HU, p = 0.02) and the temporal and occipital cortex between the measured gray matter displayed higher HU values (33 HU, p = 0.007; 31.9 HU, p = 0.008). Unfortunately, the HU value of the parietal white matter that demonstrated the largest difference between good and poor outcomes in our study was not investigated in the study. The GWR of the putamen/posterior limb of the internal capsule exhibited greater prognostic performance for prognostication (p = 0.004) than the GWR of the caudate nucleus/posterior limb of the internal capsule (p = 0.56), which was similar to the result of our study. Moreover, the HU value of the putamen and parietal white matter showed significant discrimination for prognostication in our data, and the GWR of the putamen/parietal white matter (which represents newly developing combination) must be highlighted.

The predictive capability of ONSD for ICP has been established in patients with severe traumatic brain injury [21]. In cases of brain hemorrhage, changes in ONSD due to increased ICP will be linked to poor neurological outcomes. However, in patients with cardiac arrest, where increased ICP may be present, the effectiveness of ONSD as a prognostic indicator was not demonstrated [12]. The following reasons may explain why ONSD did not reflect poor outcomes in cardiac arrest: (1) the median ICP in patients with cardiac arrest was not high (<20 mmHg) [22,23], and the time of ICP >20 mmHg was only 22 % during observation [24]; (2) in cases in which brain autoregulation is maintained, even high ICP can induce decreases in cerebral perfusion pressure (CPP) [25], and ONSD may not increase much because of low CPP; (3) in cases in which brain autoregulation is disrupted (pressure reactivity index of PRx >0.3), disrupted brain autoregulation may be switched into a normal state (4 of 7 cardiac arrest patients with disrupted autoregulation) [26]; (4) a decreased ONSD may lead to poor neurologic outcomes because the prognosis of cardiac arrest patients with low ICP was not predicted, although a high ICP indicated a poor outcome [27].

The absence of vasogenic or cytotoxic edema in brain CT is not necessarily associated with good neurological outcomes because cardiopulmonary problems before anoxic brain injury can cause poor outcomes. Thus, we compared the parameters in early CT with diffuse cortical necrosis in brain MRI and with poor neurologic outcomes. It was demonstrated that some of these parameters in early brain CT were closely correlated with the presence of diffuse cortical necrosis and poor outcomes, but the prognostic performance of these parameters was still questionable. Non-shockable rhythm (OR 13.464, p = 0.013; OR 0.14, p = 0.005 for predicting good outcomes), non-corneal reflex (OR 5.625, p = 0.001) or Glasgow coma scale (GCS) (OR 0.02, p = 0.053; OR 4.89, p = 0.044 for predicting good outcomes) on admission were much stronger predictors than GWR or ONSD [5,12,28]. A previous study reported that the risks of early neuroimaging scores consisting of sulcal effacement, partial gray-white matter effacement, total gray-white matter effacement, and deep nuclei effacement (OR 2.15, p = 0.015) were not superior to that of lactic acidosis (OR 4.2, p < 0.001) and were similar to that of the Glasgow coma scale (GCS) on admission (OR 0.43, p < 0.001) [6]. The parameters in early brain CT were not compared or analyzed with clinical characteristics in most studies. The relationship and clinical significance between the parameters in early brain CT and the outcomes should be separately considered.

## 5 Limitations

This study had several limitations. First, this retrospective observational study was conducted in a single center and had a small population. Large scale multicenter cohort study that includes a diverse range of patients should be needed to validate the results of this study because small sample sizes can lead to increased uncertainty and limited generalizability of the findings. Nevertheless, the largest number of parameters related to brain CT and MRI were included in this study. Second, confounding factors including low flow time and previous history were not analyzed comprehensively because of including small population. Potential confounders may influence the outcomes. Third, clinicians in this study were not blinded to the clinical data, and the abnormal findings of CT/MRI and knowledge may have influenced the results. Finally, the intraobserver and interobserver reliabilities of the measures were not assessed.

## 6 Conclusions

The distance of the posterior ambient cistern and the GWR of the putamen and parietal white matter in early brain CT within 24 h were promising discriminators to predict the presence of diffuse cortical necrosis and poor neurological outcome in patients with cardiac arrest. However, compared with clinical characteristics, the clinical significance was not considerable. Clinicians must deliberate on the clinical interpretation of early brain CT.

## Data availability

The datasets used and/or analyzed during the current study are available from the corresponding author on reasonable request.

## CRediT authorship contribution statement

**Jinsung Kim:** Data curation, Investigation, Writing – review & editing. **Jae Hoon Lee:** Writing – review & editing, Writing – original draft, Visualization, Supervision, Methodology, Investigation, Formal analysis, Data curation, Conceptualization.

## Declaration of competing interest

The authors declare that they have no known competing financial interests or personal relationships that could have appeared to influence the work reported in this paper.
